# The Novel Ceramide- and Phosphatidylcholine-Based Risk Score for the Prediction of New-Onset of Hypertension

**DOI:** 10.3390/jcm12247524

**Published:** 2023-12-06

**Authors:** Mohammadreza Shoghli, A. Inkeri Lokki, Mitja Lääperi, Juha Sinisalo, Marja-Liisa Lokki, Mika Hilvo, Antti Jylhä, Jaakko Tuomilehto, Reijo Laaksonen

**Affiliations:** 1 Department of Population Health, University of Helsinki, 00014 Helsinki, Finland; mohammadreza.shoghli@helsinki.fi; 2 Heart and Lung Center, Helsinki University Hospital, 00014 Helsinki, Finland; inkeri.lokki@helsinki.fi (A.I.L.); juha.sinisalo@hus.fi (J.S.); 3 Department of Bacteriology and Immunology and Translational Immunology Research Program, University of Helsinki, 00014 Helsinki, Finland; 4 Department of Pathology, University of Helsinki, 00290 Helsinki, Finland; maisa.lokki@helsinki.fi; 5 Lääperi Statistical Consulting, 02770 Espoo, Finland; 6 VTT Technical Research Centre of Finland, 02044 Espoo, Finland; mika.hilvo@vtt.fi; 7 Zora Biosciences Oy, 02620 Espoo, Finlandreijo.laaksonen@zora.fi (R.L.); 8 Population Health Unit, Finnish Institute for Health and Welfare, 00271 Helsinki, Finland; 9 Department of Public Health, University of Helsinki, 00014 Helsinki, Finland; 10 Saudi Diabetes Research Group, King Abdulaziz University, Jeddah 21589, Saudi Arabia; 11 Department of International Health, National School of Public Health, Instituto de Salud Carlos III, 28029 Madrid, Spain; 12 Finnish Cardiovascular Research Center, Tampere University Hospital, University of Tampere, 33521 Tampere, Finland

**Keywords:** ceramide, phosphatidylcholine, CERT1, CERT2, hypertension, prevalence, new-onset hypertension

## Abstract

Ceramides and other sphingolipids are implicated in vascular dysfunction and inflammation. They have been suggested as potential biomarkers for hypertension. However, their specific association with hypertension prevalence and onset requires further investigation. This study aimed to identify specific ceramide and phosphatidylcholine species associated with hypertension prevalence and onset. The 2002 FINRISK (Finnish non-communicable risk factor survey) study investigated the association between coronary event risk scores (CERT1 and CERT2) and hypertension using prevalent and new-onset hypertension groups, both consisting of 7722 participants, over a span of 10 years. Ceramide and phosphatidylcholine levels were measured using tandem liquid chromatography-mass spectrometry. Ceramide and phosphatidylcholine ratios, including ceramide (d18:1/18:0), ceramide (d18:1/24:1), phosphatidylcholine (16:0/16:0), and the ratio of ceramide (d18:1/18:0)/(d18:1/16:0), are consistently associated with both prevalence and new-onset hypertension. Ceramide (d18:1/24:0) was also linked to both hypertension measures. Adjusting for covariates, CERT1 and CERT2 showed no-longer-significant associations with hypertension prevalence, but only CERT2 predicted new-onset hypertension. Plasma ceramides and phosphatidylcholines are crucial biomarkers for hypertension, with imbalances potentially contributing to its development. Further research is needed to understand the underlying mechanisms by which ceramides will contribute to the development of hypertension.

## 1. Introduction

Hypertension is a major risk factor for cardiovascular disease (CVD) such as myocardial infarction, stroke, and heart failure [1]. The significant links between CVD risk, hypertension, the metabolic syndrome, and inflammation underline the critical need for the early identification and appropriate treatment of hypertension as a fundamental approach for improving cardiovascular health. However, the specific metabolic pathways that contribute to the development of hypertension in normotensive or prehypertensive people are not fully known.

Ceramides, involved in lipoprotein aggregation, inflammation, oxidative stress, and apoptosis, serve as potential biomarkers for cardiovascular risk in hypertensive patients and exhibit specific associations with major adverse cardio-vascular events (MACEs), highlighting their utility in risk assessment, particularly through the Coronary Event Risk Test 1 (CERT1) score [1].

The coronary event risk test 2 (CERT2) is an innovative risk score that has garnered substantial interest for its ability to predict major adverse cardiovascular events (MACE) and cardiovascular disease (CVD). Ceramides and phosphatidylcholines (PCs) are incorporated in this enhanced risk assessment method, which estimates the likelihood of future CVD incidents among people with coronary artery disease (CAD) [2].

Furthermore, there is a significant increase in the risk of cardiovascular (CV) death associated with the CERT2 risk score in the very high-risk category compared with the low-risk category. This underscores the crucial role of CERT2 as a predictive tool for adverse CV outcomes, particularly in terms of CV death [3].

Earlier investigations have presented compelling findings regarding the predictive capabilities of ceramides, specifically the CERT2 score. Notably, CERT2 scores have demonstrated their predictive power beyond traditional biomarkers such as LDL cholesterol and high-sensitivity C-reactive protein [4,5,6]. Moreover, these scores have been demonstrated to forecast cardiovascular disease (CVD) mortality in individuals with both chronic and acute coronary syndrome [2]. The utilization of the CERT2 score appears to hold promise for enhancing risk stratification in hypertensive people, potentially paving the way for a more individualized and efficient management of their cardiovascular well-being. Nonetheless, additional research is imperative to enhance and substantiate the applicability of ceramide scores in hypertensive cohorts for wider clinical use. Unknown factors pertaining to CERT scores in hypertension primarily include understanding the mechanisms and causality, determining the best ceramide profiles for various subgroups, evaluating the long-term effects, generalizability, validation in larger cohorts, clinical application, and determining the most successful intervention strategies based on CERT risk assessments. Addressing these knowledge gaps will provide a more comprehensive understanding of CERT scores in the context of hypertension.

Our study aimed at revealing novel insights into the associations between specific lipid markers, including ceramides and CERT scores, and hypertension. The novelty of our approach lies in the identification of distinct lipid species, such as ceramides, associated with prevalent and new-onset hypertension, as well as in extending the utility of CERT scores to assess the risk of hypertension. These findings contribute to our understanding of the complex interactions underlying hypertension and may have future implications for its prevention and management.

## 2. Materials and Methods

### 2.1. Study Population

The FINRISK cohorts are comprehensive, population-based surveys conducted every five years since 1972 to identify factors associated with non-communicable diseases (NCDs) and their long-term consequences [7]. In 2002, the FINRISK survey collected data from a stratified random sample of men and women aged 25–74 years from five geographic regions of Finland. The cohort consisted of 46.2% men and 53.8% women, reflecting the sex distribution in the study population [8,9]. The survey sample and methods were previously published [7,10]. In the present study, we evaluated the associations of distinct lipid species with prevalent and new-onset hypertension in people without major adverse cardiovascular events (MACE) before the study baseline (*n* = 7722). The follow-up period extended for a period of ten years.

### 2.2. Definition of Prevalent and New-Onset Hypertension

The prevalence of hypertension was determined using a combination of the baseline survey examination and registry data. It is defined based on specific criteria: systolic BP (SBP) 140 mmHg or higher and/or DBP 90 mmHg or higher. Prevalent hypertension refers to individuals who already had hypertension at the beginning of the study. 

New-onset hypertension refers to individuals who developed hypertension during the study or observation period. These individuals were initially free of hypertension but subsequently developed elevated BP during the follow-up and received BP-lowering drug treatment. New-onset hypertension during the 10-year follow-up was ascertained from the register for the reimbursement of the drug costs for hypertension maintained by the national Social Insurance Institution, using record linkage with the national personal identification number.

### 2.3. Blood Pressure Measurements

During the baseline survey, participants’ BP was measured using mercury sphygmomanometers, following established guidelines. The readings were taken from the right arm of seated participants who had rested for at least five minutes beforehand. Using the first phase of Korotkoff sounds, SBP was recorded, while DBP was recorded during the fifth phase. To ensure accuracy, BP was measured three times, and the mean value was used for this study.

### 2.4. Laboratory Measurements

The venous blood samples were centrifuged at the survey sites, and the serum was transferred to the laboratory of the Finnish Institute for Health and Welfare (THL) for cholesterol measurements. The serum was frozen immediately after separation and transferred weekly on dry ice to the laboratory for biochemical analyses. A targeted tandem liquid chromatography-mass spectrometry method was utilized to analyze the levels of four ceramides (Cer (d18:1/16:0), Cer (d18:1/18:0), Cer (d18:1/24:0), and Cer (d18:1/24:1)) and three phosphatidylcholines (PC (14:0/22:6), PC (16:0/22:5), and PC (16:0/16:0)) in participants from FINRISK 2002, as described earlier [10]. 

### 2.5. Coronary Event Test Score 1 (CERT1)

**Figure 1:**
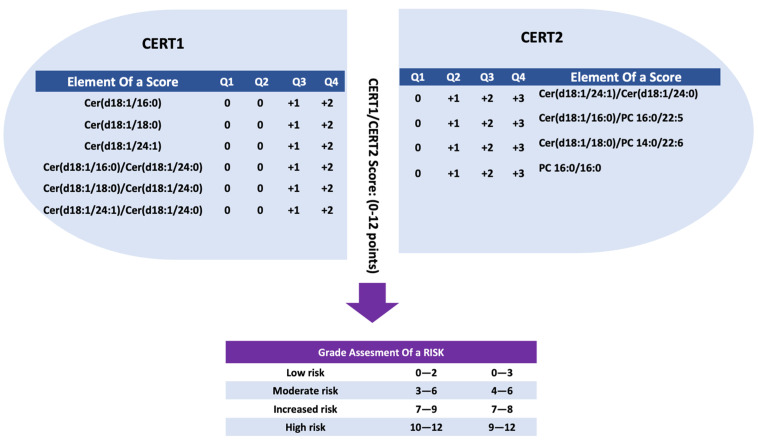
Evaluation of ceramide coronary event test scores (CERT1 and 2) risk scores and stratification by CVD risk groups. A subject’s score is determined based on which quartile he or she belongs to according to each score component. Only quartiles 3 and 4 of CERT1 give points, while CERT2 has only four components, but already the second quartile gives a risk point. Consequently, both CERT1 and CERT2 scores are on the same scale (0–12). Based on these factors, CVD risk categories are determined for each subject. Cer: Ceramide, PC: phosphatidylcholine.

CERT1 is a calculation that evaluates ceramide concentrations and ratios by comparing the levels of Cer (d18:1/16:0), Cer (d18:1/18:0), and Cer (d18:1/24:1) to Cer (d18:1/24:0). This analysis provides insights into the specific ceramide components and their ratios [11]. Participants with concentrations or ratios in the fourth quartile received two points, those in the third quartile received one point, and those in the first and second quartiles received zero points. The CERT1 score ranges from 0 to 12 [11] and is shown in Figure 1.

### 2.6. Coronary Event Test Score 2 (CERT2)

To enhance CERT1 and incorporate the prognostic significance of PCs in relation to CVD events [10,12], a subsequent development called CERT2 was introduced earlier. CERT2 consists of one ceramide/ceramide ratio (Cer (d18:1/24:1)/(d18:1/24:0)), two ceramide/PC ratios (Cer (d18:1/16:0)/PC (16:0/22:5) and Cer (d18:1/18:0)/(PC 14:0/22:6)), and a single PC (PC 16:0/16:0). The scoring system for CERT2 ranges from 0 to 12 points, depending on the summed points obtained by the individual (Figure 1). 

### 2.7. Statistical Analyses

**Figure 2:**
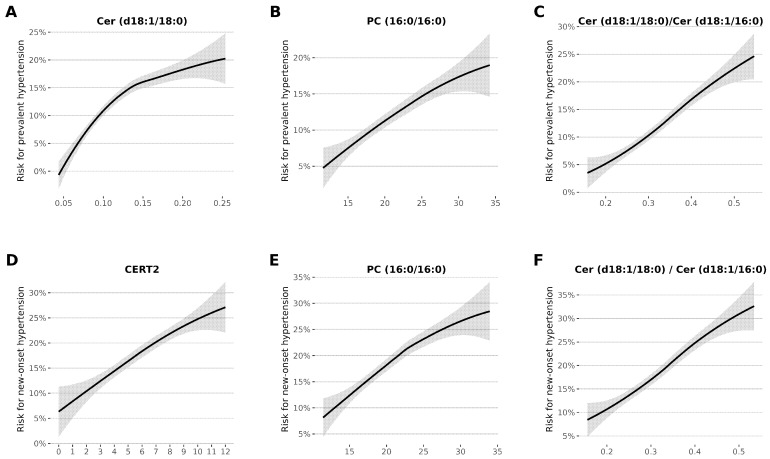
This figure illustrates ceramide associations with both prevalent and new-onset hypertension, emphasizing the key factors influencing hypertension development. The solid line represents the fitted loess regression line, and the shaded area corresponds to the 95% confidence interval band. Values below the 1st percentile and above the 99th percentile are winsorized to prevent long uninformative tails in lipids and lipid ratios. Figures (**A**–**C**) demonstrate prevalent hypertension, while (**D**–**F**) depict new-onset hypertension.

The baseline characteristics of the cohorts were presented with means and standard deviations (SD) for continuous variables and percentages for categorical variables. The study employed cox proportional hazard models with age during the study as the timescale and logistic regression to calculate hazard ratios, odds ratios, and their corresponding confidence intervals, both before and after adjusting for covariates. Logistic regression was used to explore the association between CERT scores/components and prevalent hypertension. Univariable and multivariable Cox regression models were also utilized to investigate the association between CERT scores and components and new-onset hypertension, with age as the time scale. All analyses were performed with R-software version 4.3.1, using the survival-package for cox regression models and ggplot2-package for creating the plots. We considered *p*-values below 0.05 to be significant. In Figure 2, we employed the loess (locally estimated scatterplot smoothing) method to analyze the risk of prevalent and new-onset hypertension and generated risk plots for selected variables.

The FINRISK 2002 risk charts were generated by utilizing the median of the risk score category points for the CERT2 group. In our ceramide study, multivariable regression models were fitted using three combination models of covariates named M1, M2, and M3. Model 1 included baseline age and sex, while Model 2 included Model 1 along with serum total cholesterol (TC), high-density lipoprotein (HDL) cholesterol, and low-density lipoprotein (LDL) cholesterol. Model 3 included Model 2 along with additional baseline variables such as body mass index (BMI), current smoking status, prevalent diabetes, and lipid-lowering drug treatment.

### 2.8. Statistical Software and Packages

R Software: For statistical analyses, we utilized R software, version 4.3.1 (Vienna, Austria.You can access R at the <https://www.R-project.org/>, accessed on 29 September 2023.

Survival Package: To conduct survival analysis in R, we utilized the “survival” package developed by Therneau T (2023), version 3.5-5. Additional information can be found on the Comprehensive R Archive Network (CRAN) at <https://CRAN.R-project.org/package=survival>, accessed on 29 September 2023.

ggplot2 Package: For creating compelling data visualizations, we relied on the “ggplot2” package by H. Wickham (2016) (https://ggplot2.tidyverse.org/).

## 3. Results

### 3.1. Baseline Characteristics

Table 1 presents an overview of the key characteristics related to the study participants. As anticipated, a majority of clinical risk factors such as age, BMI, waist circumference, serum triglycerides (TG), TC and HDL, C-reactive protein (CRP), and history of diabetes mellitus showed significant differences between individuals with and without hypertension. Men had a higher frequency of hypertension than women in the prevalent hypertension group, but not in the new-onset hypertension group.

### 3.2. Association of CERT1 and CERT2 Scores with Prevalent and New-Onset Hypertension

The univariable logistic analyses demonstrated a statistically significant association between both CERT1 and CERT2 scores and prevalent hypertension. The odds ratio (OR) for CERT1 scores was 1.42 (95% CI: 1.32–1.52, *p* < 0.001), while the OR for CERT2 scores was 1.36 (95% CI: 1.26–1.46, *p* < 0.001) (Table 2). The results of the multivariable regression analyses revealed that the association between CERT1 score, and prevalent hypertension remained significant after adjusting for covariates in Model 1 (age and sex) and Model 2 (additionally for serum lipids). However, this significance was no longer observed in Model 3 (adjusting further for BMI, smoking, diabetes, and lipid-lowering drug treatment). In contrast, no significant association between the CERT2 score and prevalent hypertension was found in the multivariable regression analysis after adjusting for covariates in each of the three models.

For new-onset hypertension, univariable logistic analyses showed a statistically significant association between both CERT1 and CERT2 scores. The hazard ratio (HR) for the CERT1 score was 1.16 (95% CI: 1.09–1.23, *p* < 0.001), and for the CERT2 score, 1.16 (95% CI: 1.10–1.23, *p* < 0.001) (Table 2). The results of the multivariable regression analyses revealed that the association between both CERT1 and CERT2 scores, and new-onset hypertension remained significant after adjusting for covariates in Model 2 and Model 3, although it was borderline for CERT1 in Model 3. Model 1 was not included, since we used age at the baseline and age at new-onset hypertension as follow-up times.

### 3.3. Association between Lipid Species and Prevalent and New-Onset Hypertension

Associations between measured lipid species and their distinct ratios with the prevalence of hypertension were evaluated. In the unadjusted model, all measured lipid species, except for Cer (d18:1/16:0)A/Cer (d18:1/24:0), showed a significant association with prevalent hypertension. In all models, Cer (d18:1/24:1)/Cer (d18:1/24:0) showed a significant association with prevalent hypertension. The highest odds ratio (OR) of 1.63 (95% CI 1.52–1.76) was observed for the Cer (d18:1/18:0)/Cer (d18:1/16:0) ratio, and this association remained significant even after all adjustments. In addition, the study revealed that Cer (d18:1/18:0) consistently played a significant predictive role in all three models (Table 3).

Significant associations were observed between most of the lipid species and new- onset hypertension (Table 3). Cer (d18:1/18:0), either alone or in ratios, such as Cer (d18:1/18:0)/PC (14:0/22:6), consistently appearing as significant predictors of new-onset hypertension in all adjustment models. Additionally, PC (16:0/16:0) emerged as another significant lipid predictor of new-onset hypertension (Table 3).

### 3.4. Factors Linked to Prevalent and New-Onset Hypertension

To investigate factors contributing to hypertension, our analysis reveals significant associations with both prevalent and new-onset hypertension. Prevalent hypertension is linked to factors including Cer (d18:1/18:0), PC (16:0/16:0), and the Cer (d18:1/18:0)/Cer (d18:1/16:0) ratio. New-onset hypertension, on the other hand, shows associations with specific factors, such as CERT2, PC (16:0/16:0), and the Cer (d18:1/18:0)/Cer (d18:1/16:0) ratio. These findings highlight elements influencing both the presence and onset of hypertension (Figure 2).

### 3.5. A Closer Look at the Effect of Sex on Baseline Characteristics and Regression Models

In this study, we conducted a comparative analysis of baseline characteristics between male and female participants (see Supplementary Table S1). To provide a detailed examination of sex-specific results, we conducted separate regression analyses for male and female subgroups. Additionally, we investigated potential interactions between sex and the biomarkers. The results for these analyses are available in Supplementary Tables S2 and S3. Notably, the majority of measured lipid concentrations were higher in males when compared to females. Furthermore, we observed an association between CERT2 and hypertension in both genders.

## 4. Discussion

CERT1 and CERT2 scores have earlier been shown to be very good prognostic markers for CVD death, myocardial infarction, stroke, and heart failure [10]. In the present study, we evaluated the associations between these two scores and their components with hypertension. Both risk scores were associated with prevalent and new-onset hypertension. However, adjustment for covariates attenuated the significance of these associations, especially for prevalent hypertension. After the full adjustment model, only CERT2 showed a significant predictive value for new- onset hypertension, while CERT1 became borderline significant. These data corroborate that lipid metabolism likely plays a role in the development of hypertension [13]. 

In 2017, Wang et al. found that ceramides, including Cer (d18:1/16:0), Cer (d18:1/18:0), Cer (d18:1/24:0), and Cer (d18:1/24:1), were associated with an increased risk of CVD. Their study revealed that median concentrations of these ceramides increased gradually from healthy individuals to people with hypertension and a low-to-medium CVD risk, and further to those with a high CVD risk. Interestingly, the median concentration of Cer (d18:1/16:0) was 13% higher in patients with CVD events compared to those without evidence of CVD [14]. Recently, a novel ceramide-based score for predicting major adverse cardiac events (MACE) in hypertensive individuals called CERT-HBP has been introduced [1]. The score incorporates the plasma levels of Cer (d18:1/16:0), Cer (d18:1/24:1), and their ratios to Cer (d18:1/24:0) and Cer (d18:1/22:0). These ceramide species were found to be highly significant in predicting MACE in people with hypertension and high CVD risk [1]. Isolated nocturnal hypertension (INH) has been found to be associated with Cer (18:1/16:0), indicating that Cer (18:1/16:0) could be a potential biomarker for the diagnosis of INH [15]. In our study, the data for INH were not available.

The present study revealed that PC can serve as a significant predictor of hypertension, in addition to ceramides. Specifically, PC (16:0/16:0) emerged as a predictive factor for both prevalent and new-onset hypertension, while PC (14:0/22:6) was associated with prevalent hypertension. These findings align with those of a prior study that identified two PC species (PC 32:1 or PC (16:0/16:0) and PC 40:5), along with six phosphatidylethanolamines, as predictors of new-onset hypertension [16].

Moreover, our study observed higher lipid concentrations in males, which may have clinical relevance in terms of treatment. However, it is important to note that these observations have a limited impact on the CERT2 score’s ability to assess the association between lipid concentrations and hypertension. While sex-specific differences in lipid profiles are noteworthy, the overall predictive power of CERT2 in hypertension remains a crucial focus of our investigation.

While the precise mechanisms linking ceramides to hypertension remain unclear, the association between ceramide Cer (d18:1/18:0) and features of the metabolic syndrome is well established. Cer (d18:1/18:0) serves as a robust biomarker, reflecting cardiovascular damage and demonstrating predictive value for CVD. Elevated levels of Cer (d18:1/18:0) are strongly implicated in the development of insulin resistance, dyslipidemia, and cardiovascular pathologies. Notably, inhibiting ceramide synthesis has shown promising outcomes in reducing ventricular remodeling and preventing cardiac failure. Thus, Cer (d18:1/18:0) emerges as a pivotal biomarker and a potential therapeutic target for improving clinical outcomes in individuals with the metabolic syndrome [17]. In our study, we identified the Cer (d18:1/24:1)/Cer (d18:1/24:0) ratio as significant in both prevalent and new-onset hypertension. Notably, Cer (d18:1/24:0) emerged as significant in new-onset hypertension. These findings align with a previous study on stenosis and acute coronary syndromes (ACS), where Cer (d18:1/16:0), Cer (d18:1/24:0), and the Cer (d18:1/24:1)/Cer (d18:1/24:0) ratio were also identified as significant factors. This consistent pattern underscores the relevance of ceramides as crucial biomarkers across various cardiovascular conditions [18].

It has been shown that ceramides have the potential to activate Ras homolog family member A (RhoA)/Rho-kinase (ROCK) signaling, leading to vasoconstriction and hypertension in animal models similar to angiotensin II [13].

Both angiotensin II and ceramides may contribute to hypertension by increasing oxidative stress in vascular cells. This, in turn, can lead to endothelial dysfunction and impaired vasodilation [19,20]. On the other hand, recent studies have shown that genetic variations in the metabolic sphingolipid network are associated with changes in blood pressure, indicating a potential role for sphingolipids in the development of hypertension [13]. Smooth muscle contraction is regulated by sphingosine-1-phosphate (S1P) through RhoA activation. Out of the 353 different metabolic sphingolipid gene exon variants, 34 are associated with DBP, and 40 are linked to SBP [13]. Sphingosine 1-kinase (SPHK1) and ceramidase (ASAH1) work together to produce ceramide. This gene pair has been linked to hypertension, providing further evidence of S1P (sphingosine 1-phosphate) and RhoA signaling involvement [21,22].

The protein kinase B (AKT/PKB) pathway, a complex signaling network involved in cellular processes such as cell survival, growth, and metabolism, is a promising area of research for understanding hypertension [23]. The activation of AKT/PKB can lead to vasodilation through nitric oxide synthase (NOS) activation and increased nitric oxide (NO) production [24,25]. Furthermore, this pathway plays a role in regulating ceramide metabolism and hypertension, with AKT activation upregulating ceramide synthase 1 (CerS1) and increasing ceramide production [26].

TXNIP (thioredoxin-interacting protein) emerges as a novel biomarker for ischemia, CVD, and hypertension [27]. Ceramides, by upregulating TXNIP and stimulating the NOD-like receptor family and pyrin domain-containing 3 inflammasome (NLRP3), impair Pulmonary Microvascular Endothelial Cell (PMVEC) barrier function and promote the production of pro-inflammatory mediators like IL-1 (interleukin-1) [28]. Clinical genomic studies have linked variations in TXNIP transcript, elevated BP, arterial stiffness, and coronary heart disease, suggesting that imbalanced ceramide levels and subsequent TXNIP dysregulation may contribute to hypertension [29,30]. Further research is needed to elucidate the specific function, mode of action, and impact of TXNIP on hypertension and ceramide levels.

Our findings indicate a significant association between Cer (d18:1/18:0) and hypertension, as well as the Cer (d18:1/18:0)/Cer (d18:1/16:0) ratio. Importantly, previous research has also demonstrated connections between Cer (d18:1/18:0) and Cer (d18:1/16:0) ratios with type 2 diabetes, further emphasizing the link between Cer (d18:1/18:0) and the metabolic syndrome [31]. This is consistent with prior evidence indicating that the Cer (18:1/18:0)/Cer (18:1/16:0) ratio was predictive of incident diabetes [32]. These results suggest that Cer (d18:1/18:0) and Cer (d18:1/16:0) play a significant role in the development and manifestation of hypertension and may have implications in the context of metabolic diseases.

L-serine, an amino acid, plays a crucial role in cellular metabolism as it serves as a foundational building block for vital compounds, including phosphatidylserine (PS), ceramides, and other sphingolipids [33]. Recent evidence suggests a potential connection between L-serine deficiency and hypertension. While initially associated with conditions like diabetic neuropathy, which affects both limbs (peripheral neuropathy) and internal organs (autonomic neuropathy), the impact of L-serine deficiency on blood pressure regulation has gained attention. This suggests that L-serine, as an essential amino acid, may play a role in modulating blood pressure [33]. Additionally, CerS5, an isoform of ceramide synthase, is intricately involved in the synthesis of ceramide species, particularly its capacity to generate C16-ceramide, a well-known pro-apoptotic ceramide [34]. Notably, genome-wide association studies (GWAS) have established a connection between CerS5 and hypertension, shedding light on its potential significance in cardiovascular health and suggesting that CerS5′s impact on ceramide species may have implications for hypertension [35].

There are limitations in our study: the study did not adjust for unknown and/or unavailable potential confounding variables in some statistical analyses. Also, our study’s generalizability may be limited due to its focus on a single, although randomly selected, population cohort of European ancestry. Further research in diverse populations is needed to validate and extend our findings. Moreover, as an observational study, our results highlight associations rather than establishing causality, emphasizing the need for additional investigations. Furthermore, the utility of ceramide scores in hypertensive populations for broader clinical applications requires refinement and validation, presenting a critical avenue for future research.

## 5. Conclusions

This study emphasizes the connection between ceramides and hypertension, underscoring the need for further research to uncover the underlying mechanisms and identify potential new targets for hypertension treatment, particularly in the context of cardiovascular healthcare. Our findings highlight the clinical relevance of utilizing CERT scores in assessing the risk of hypertension, offering valuable insights into the intricate nature of the metabolic syndrome. This approach provides healthcare professionals with a more detailed strategy for the tailored treatment and prevention of hypertension within the broader scope of cardiovascular healthcare.

## Figures and Tables

**Table 1:** Selected baseline characteristics of participants with and without prevalent and new-onset hypertension. Values are means or percentages.

**Study on Prevalent Hypertension**			
**Variable**	**People without Hypertension at Baseline (*n* = 6847)**	**People with Hypertension at Baseline (*n* = 875)**	***p*** **-Value**
Age (years)	46.7 (12.9)	59.2 (8.8)	<0.001
Men *n* (%)	3157 (46.1%)	462 (52.8%)	<0.001
BMI (Kg/m^2^)	26.5 (4.5)	30.1 (4.9)	<0.001
Waist circumference (cm)	88.2 (13.1)	99.0 (13.1)	<0.001
Serum triglycerides (mmol/L)	1.4 (0.9)	1.8 (1.2)	<0.001
Serum total cholesterol (mmol/L)	5.6 (1.1)	5.7 (1.0)	0.002
Serum HDL (mmol/L)	1.5 (0.4)	1.4 (0.4)	<0.001
Serum CRP (mg/dL)	2.4 (4.9)	3.8 (7.9)	<0.001
Current smoking	1840 (26.9%)	132 (15.1%)	<0.001
Systolic BP (mmHg)	133 (19.5)	162 (19.7)	<0.001
Diastolic BP (mmHg)	78 (11.1)	84 (10.4)	<0.001
Diabetes mellitus	290 (4.2%)	159 (18.2%)	<0.001
History of lipid-lowering drug treatment	355 (5.2%)	220 (25.1%)	<0.001
History of BP-lowering drug treatment	330 (4.8%)	793 (90.6%)	<0.001
CERT1	4.4 (3.2)	5.5 (3.2)	<0.001
CERT2	5.9 (2.4)	6.7 (2.3)	<0.001
Death during the follow-up	631 (9.2%)	217 (24.8%)	<0.001
**Study on New-onset Hypertension**			
**Variable**	**People without Hypertension at the Baseline (*n* = 5621)**	**People with Hyper-tension at the Baseline (*n* = 1225)**	***p*** **-Value**
Age (years)	45.0 (12.7)	54.3 (10.9)	<0.001
Men *n* (%)	2550 (45.4%)	606 (49.5%)	0.010
BMI (Kg/m^2^)	26.0 (4.2)	28.8 (4.9)	<0.001
Waist circumference (cm)	86.8 (12.5)	94.8 (14.0)	<0.001
Serum triglycerides (mmol/L)	1.3 (0.9)	1.7 (1.1)	<0.001
Serum total cholesterol (mmol/L)	5.5 (1.1)	5.8 (1.1)	<0.001
Serum HDL (mmol/L)	1.5 (0.4)	1.5 (0.4)	<0.001
Serum CRP (mg/dL)	2.2 (4.7)	3.3 (5.6)	<0.001
Current smoking	1545 (27.5%)	294 (24.0%)	0.014
Systolic BP (mean)	129 (16.9)	150(21.1)	<0.001
Diastolic BP (mean)	77 (10.4)	86 (11.3)	<0.001
Diabetes mellitus	177 (3.1%)	112 (9.1%)	<0.001
Lipid-lowering drug treatment	225 (4.0%)	130 (10.6%)	<0.001
BP-lowering drug treatment	121 (2.2%)	209 (17.1%)	<0.001
CERT1	4.2 (3.2)	5.3 (3.3)	<0.001
CERT2	5.8 (2.4)	6.5 (2.3)	<0.001
Death during the follow-up	451 (8.0%)	179 (14.6%)	<0.001

BMI: Body mass index; HDL: high-density lipoprotein cholesterol; CRP: c-reactive protein; BP: blood pressure; CERT1: coronary event test score 1; CERT2: coronary event test score 2.

**Table 2:** Univariable and multivariable regression analyses for CERT1 and CERT2 score considering prevalent and new onset hypertension.

Variables		Study on Prevalent Hypertension		Study on New-Onset Hypertension	
		OR (95% CI)	*p* Value	HR (95% CI)	*p* Value
CERT1	(Unadjusted)	1.42 (1.32–1.52)	<0.001	1.16 (1.10–1.23)	<0.001
CERT2	(Unadjusted)	1.36 (1.26–1.46)	<0.001	1.16 (1.09–1.23)	<0.001
CERT1	(Adjusted for M1)	1.12 (1.04–1.21)	0.002	-	-
CERT2	(Adjusted for M1)	1.06 (0.98–1.15)	0.140	-	-
CERT1	(Adjusted for M2)	1.11(1.02–1.20)	0.013	1.12 (1.06–1.19)	<0.001
CERT2	(Adjusted for M2)	1.05(0.96–1.13)	0.280	1.13 (1.06–1.20)	<0.001
CERT1	(Adjusted for M3)	1.03(0.94–1.12)	0.550	1.05 (0.98–1.11)	0.160
CERT2	(Adjusted for M3)	1.04(0.96–1.14)	0.350	1.09 (1.02–1.16)	0.009

OR: Odds ratio, HR: hazard ratio, CI: confidence interval, CERT: coronary event risk test; HDL: high-density lipoprotein cholesterol, LDL: low-density lipoprotein cholesterol; Model 1: (age + sex); Model 2: (M1 + serum total cholesterol + HDL+ LDL); Model 3: (M2 + BMI + Current Smoking + Diabetes Mellitus + History of Lipid-lowering Drug Treatment); In the study on new-onset hypertension, Model 1 is not included, we used age as follow-up time, and sex was included as a stratifying variable in the models. Bolds: Significance value for *p* < 0.05. Prevalent hypertension is defined as current treatment with blood-pressure-lowering drugs or systolic BP > 140 mmHg and/or diastolic BP > 90 mmHg. New-onset hypertension is defined as the initiation of new blood-pressure-lowering drug treatment after the baseline.

**Table 3:** Univariable and multivariable regressi1on for CERT score components in the study on prevalent and new-onset hypertension. Statistically significant *p*-values (<0.05) are shown in bold.

**Study on Prevalent Hypertension**								
	**Unadjusted**		**Adjustment for Model 1**		**Adjustment for Model 2**		**Adjustment for Model 3**	
**Components**	**OR (95% CI)**	***p* Value**	**OR (95% CI)**	***p* Value**	**OR (95% CI)**	***p* Value**	**OR (95% CI)**	***p* Value**
Cer (d18:1/16:0)	1.22 (1.14–1.31)	<0.001	0.98 (0.90–1.06)	0.58	0.98 (0.89–1.09)	0.75	1.00 (0.90–1.11)	0.95
Cer (d18:1/18:0)	1.61 (1.50–1.73)	<0.001	1.30 (1.20–1.41)	<0.001	1.37 (1.25–1.51)	<0.001	1.14 (1.03–1.26)	0.012
Cer (d18:1/24:0)	1.22 (1.13–1.31)	<0.001	1.03 (0.95–1.12)	0.43	1.09 (0.98–1.21)	0.10	1.06 (0.95–1.18)	0.32
Cer (d18:1/24:1)	1.49 (1.39–1.60)	<0.001	1.16 (1.07–1.26)	0.0027	1.23 (1.11–1.37)	0.0091	1.18 (1.06–1.31)	0.0030
PC (14:0/22:6)	1.33 (1.24–1.44)	<0.001	1.09 (1.00–1.18)	0.039	1.16 (1.06–1.27)	0.0019	1.1 (1.00–1.24)	0.049
PC (16:0/16:0)	1.36 (1.27–1.46)	<0.001	1.04 (0.97–1.13)	0.29	1.21 (1.09–1.33)	0.0030	1.2 (1.08–1.34)	0.0064
PC (16:0/22:5)	1.14 (1.06–1.22)	0.0033	1.05 (0.97–1.13)	0.22	1.15 (1.05–1.27)	0.002	1.05 (0.95–1.16)	0.34
Cer (d18:1/16:0)/Cer (d18:1/24:0) ratio	0.98 (0.91–1.05)	0.51	0.94 (0.87–1.01)	0.10	0.93 (0.86–1.00)	0.057	0.96 (0.89–1.04)	0.36
Cer (d18:1/18:0)/Cer (d18:1/24:0) ratio	1.49 (1.38–1.60)	<0.001	1.32 (1.22–1.43)	<0.001	1.26 (1.16–1.36)	<0.001	1.09 (1.00–1.19)	0.060
Cer (d18:1/24:1)/Cer (d18:1/24:0) ratio	1.35 (1.25–1.45)	<0.001	1.16 (1.07–1.25)	0.0001	1.10 (1.02–1.19)	0.012	1.09 (1.01–1.19)	0.031
Cer (d18:1/16:0)/PC (16:0/22:5) ratio	1.07 (1.00–1.15)	0.045	0.94 (0.87–1.01)	0.094	0.9 (0.83–0.97)	0.0071	0.97 (0.89–1.05)	0.47
Cer (d18:1/18:0)/PC (14:0/22:6) ratio	1.08 (1.00–1.15)	0.039	1.1 (1.02–1.19)	0.012	1.06 (0.98–1.15)	0.17	1.00 (0.91–1.09)	0.93
Cer (d18:1/18:0)/Cer (d18:1/16:0) ratio	1.63 (1.52–1.76)	<0.001	1.46 (1.35–1.58)	<0.001	1.40 (1.29–1.52)	<0.001	1.14 (1.05–1.25)	0.0027
**Study on New-onset Hypertension**								
	**Unadjusted**		**Adjustment for Model 1**		**Adjustment for Model 2**		**Adjustment for Model 3**	
**Components**	**HR (95% CI)**	***p* Value**	**HR (95% CI)**	***p* Value**	**HR (95% CI)**	***p* Value**	**HR (95% CI)**	***p* Value**
Cer (d18:1/16:0)	1.11 (1.05–1.18)	0.0005	―	―	1.08 (1.00–1.16)	0.055	1.07 (1.00–1.16)	0.059
Cer (d18:1/18:0)	1.29 (1.22–1.37)	<0.001	―	―	1.29 (1.20–1.38)	<0.001	1.13 (1.05–1.21)	0.0016
Cer (d18:1/24:0)	1.12 (1.06–1.19)	0.0014	―	―	1.10 (1.02–1.19)	0.011	1.09 (1.01–1.18)	0.027
Cer (d18:1/24:1)	1.19 (1.11–1.26)	<0.001	―	―	1.16 (1.07–1.25)	0.0019	1.1 (1.02–1.19)	0.013
PC (14:0/22:6)	1.01 (0.95–1.07)	0.87	―	―	0.99 (0.92–1.05)	0.69	0.97 (0.90–1.04)	0.36
PC (16:0/16:0)	1.10 (1.04–1.17)	0.0016	―	―	1.15 (1.07–1.24)	0.0003	1.12 (1.04–1.21)	0.0031
PC (16:0/22:5)	1.06 (1.00–1.12)	0.046	―	―	1.07 (1.00–1.15)	0.062	1.04 (0.97–1.11)	0.29
Cer (d18:1/16:0)/Cer (d18:1/24:0) ratio	0.97 (0.92–1.03)	0.31	―	―	0.97 (0.92–1.03)	0.37	0.98 (0.93–1.04)	0.57
Cer (d18:1/18:0)/Cer (d18:1/24:0) ratio	1.22 (1.15–1.29)	<0.001	―	―	1.18 (1.11–1.25)	<0.001	1.05 (0.99–1.12)	0.099
Cer (d18:1/24:1)/Cer (d18:1/24:0) ratio	1.06 (1.00–1.12)	0.055	―	―	1.04 (0.98–1.10)	0.19	1.01 (0.95–1.07)	0.77
Cer (d18:1/16:0)/PC (16:0/22:5) ratio	1.04 (0.98–1.10)	0.18	―	―	1 (0.94–1.06)	0.95	1.02 (0.96–1.08)	0.54
Cer (d18:1/18:0)/PC (14:0/22:6) ratio	1.17 (1.11–1.24)	<0.001	―	―	1.15 (1.08–1.22)	0.00007	1.09 (1.02–1.16)	0.0098
Cer (d18:1/18:0)/Cer (d18:1/16:0) ratio	1.29 (1.22–1.37)	<0.001	―	―	1.24 (1.17–1.32)	<0.001	1.08 (1.02–1.15)	0.015

Ceramides, PC (phosphatidylcholine), and OR (odds ratio) are important terms in our study. We define prevalent hypertension as current treatment with blood-pressure-lowering drugs or systolic BP > 140 mmHg and/or diastolic BP > 90 mmHg. New-onset hypertension is defined as the initiation of new blood-pressure-lowering drug treatment after the baseline. We denote statistically significant *p*-values (<0.05) in bold. Our analysis involves three models: Model 1 (Age + Sex), Model 2 (M1 + Serum Total Cholesterol + HDL + LDL), and Model 3 (M2 + BMI + Current Smoking + Diabetes Mellitus + History of Lipid-lowering Drug Treatment). In the case of new-onset hypertension, Model 2 replaces Model 1, and age is used as the follow-up time. We also include sex as a stratifying variable in the models.

## Data Availability

The data are available through the THL Biobank.

## Supplementary Materials

The following supporting information can be downloaded at: https://www.mdpi.com/article/10.3390/jcm12247524/s1, Table S1: Comparison of Baseline Characteristics between Study Subjects (Males vs Females). Table S2: Univariable and Multivariable Regression Analysis of CERT Scores and Their Components in the Context of Hypertension Prevalence, Stratified by Sex with Consideration of Female-Specific Interaction. Table S3: Univariable and Multivariable Regression Analysis of CERT Scores and Their Components in the Context of New Onset Hypertension, Stratified by Sex with Consideration of Female-Specific Interaction.
